# Platelet to high-density lipoprotein cholesterol ratio predicts clinical outcomes after acute ischemic stroke: a prospective cohort study

**DOI:** 10.3389/fneur.2026.1851022

**Published:** 2026-06-30

**Authors:** Xuan Sun, Haochen Sun, Zhijia Tang, Xinyang Qi, Xian Wang, Xiaoyin Wang

**Affiliations:** 1 Department of Clinical Laboratory, The Affiliated Brain Hospital of Nanjing Medical University, Nanjing, China; 2 Department of Neurology, The Affiliated Brain Hospital of Nanjing Medical University, Nanjing, China; 3 Department of Medicine, Tongling Polytechnic College, Tongling, China; 4 Department of Health Management & Institute of Health Management, Sichuan Provincial People’s Hospital, University of Electronic Science and Technology of China, Chengdu, China

**Keywords:** acute ischemic stroke, all-cause death, platelet to high-density lipoprotein cholesterol ratio, poor functional outcome, prognostic biomarker, stroke recurrence

## Abstract

**Background:**

The platelet/high-density lipoprotein cholesterol ratio (PHR), a marker of hypercoagulable states and disordered lipid metabolism, has been confirmed as a predictor of cardiovascular disease. However, the effects of PHR on the prognosis of acute ischemic stroke (AIS) remain unknown. We aimed to assess the associations of PHR with the risk of clinical outcomes in patients with AIS.

**Methods:**

This prospective observational study included 820 patients (median age, 68 years; female, 34.6%; median NIHSS at admission, 3) with AIS. The median time from symptom onset to admission was 2 days (interquartile range [IQR], 0–4), and from admission to blood sampling was 15 h (IQR, 12–19). PHR was calculated as platelet count (PC; 10^9^ cells/L)/HDL-C (mmol/L) at admission. PHR was analyzed both as a continuous variable and in tertile form (tertile 1-tertile 3). To analyze the associations between PHR and clinical outcomes including all-cause death, stroke recurrence and poor functional outcome at 3 months, 6 months and 1 year, we used multivariable Cox and logistic regression, Kaplan–Meier survival curves, restricted cubic splines, subgroup analysis, concordance statistic (C-statistic), net reclassification index (NRI), and integrated discrimination improvement index (IDI).

**Results:**

The median PHR was 202.155 (IQR, 153.120–262.365). Kaplan–Meier survival curves identified tertile 3 as the group with the highest risk for all-cause death and stroke recurrence. After adjustment, multivariable Cox regression (tertile 1 as reference) showed that the highest PHR tertile 3 was associated with increased risk for both all-cause death and stroke recurrence across all three follow-up intervals (3 months, 6 months and 1 year). In parallel, multivariable logistic regression (tertile 1 as reference) showed that tertile 3 was associated with a greater likelihood of poor functional outcome across the same three time points. Continuous PHR showed a positive dose–response relationship with clinical outcomes. Subgroup analysis revealed significant interactions of age (*p* < 0.05) with PHR for all-cause death, and of BMI (*p* < 0.05) with PHR for mRS 3–6. A basic model’s predictive ability was strengthened by the addition of PHR (C-statistic, NRI, IDI).

**Conclusion:**

A higher PHR level in patients with AIS is strongly associated with an increased risk of all-cause death, stroke recurrence and poor functional outcome. As a valuable predictive biomarker, PHR may provide a simple and effective tool for predicting clinical outcomes in patients with AIS.

## Graphical abstract

**Figure:**
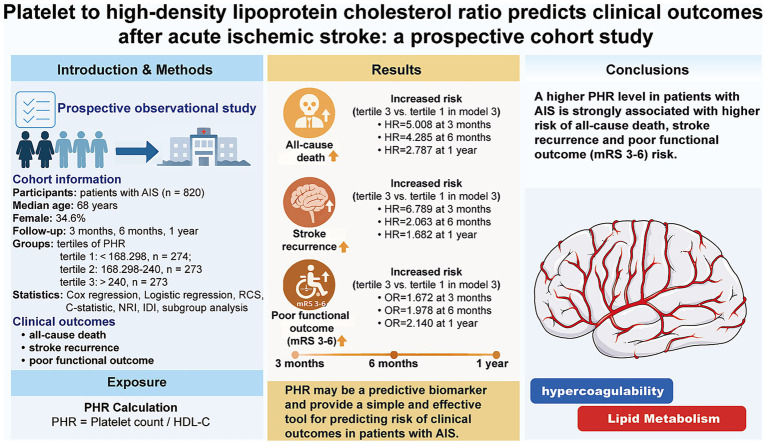
A higher PHR level in patients with AIS predicts increased risks of all-cause death, stroke recurrence, and poor functional outcome.

## 1 Introduction

Acute ischemic stroke (AIS) arises from sudden or gradual occlusion of the cerebral arteries, representing a medical emergency (1). Globally, stroke ranked as the third major cause of mortality and disability in 2021 (2, 3). The high incidence and mortality of AIS have become a serious public health problem worldwide (4). Given the strict time window and limited eligibility for mechanical thrombectomy and intravenous thrombolysis, many patients with AIS still receive conservative medical treatment (5, 6). Therefore, improving prognostic assessment, facilitating the early recognition of high-risk individuals, and implementing timely interventions based on robust predictors may improve outcomes after AIS (7, 8).

The pathological signature of ischemic stroke involves a set of closely interrelated neuropathological processes, among which a strong and persistent inflammatory response contributes to the aggravation of brain injury (9). Inflammation can increase stroke risk by promoting atherosclerosis, damaging vascular endothelial function, and enhancing thrombosis (1, 10, 11). Decades of research have identified the anti-atherogenic functions of high-density lipoprotein cholesterol (HDL-C), including the promotion of reverse cholesterol transport, along with both anti-inflammatory and antioxidant properties. Therefore, HDL-C is regarded as both a marker of cardiovascular disease (CVD) risk and a potential therapeutic target (12, 13). Moreover, the onset and progression of AIS are often accompanied by complex hematological changes, such as activation of platelets (PLTs), endothelial cells, and the fibrinolytic system (14). PLTs contribute to both hemostasis and the promotion of coagulation (5). Further exacerbation of ischemia results from an imbalance between coagulation and fibrinolytic systems. Thrombi mainly contain varying amounts of fibrin, PLTs, red blood cells, von Willebrand factor (vWF), and neutrophil extracellular traps (NETs) (15, 16). These findings suggest that the combination of PLT with HDL-C levels may help estimate the risk of poor prognosis after AIS. Building on these respective functions of platelets and HDL-C, PLT/HDL-C ratio (PHR) has been proposed as a biomarker that reflects both abnormal hypercoagulability and lipid metabolism disorders. Notably, PHR has shown some associations with CVDs and metabolic diseases in clinical studies (17–20). Previous studies demonstrated that elevated PHR levels correlate with more severe coronary artery disease (CAD) and that this ratio may additionally serve as a robust biomarker for metabolic syndrome and heightened atherothrombotic risk (17, 21). Other lipid-related ratios have also demonstrated prognostic value in AIS. For instance, the atherogenic index of plasma (AIP, AIP = log [TG/HDL-C]), has been reported as an independent predictor of 1-month mortality in AIS patients, with lower AIP associated with higher mortality risk (22). These findings suggest that lipid-related ratios may offer potential prognostic value beyond traditional single lipid parameters.

We hypothesized that PHR could better integrate the combined impact of hypercoagulable states and lipid metabolic disorders that underlie both cardiovascular and cerebrovascular disease. However, evidence regarding PHR’s association with clinical outcomes in AIS patients is lacking. We therefore collected PLT, HDL-C, and other clinical data to investigate how PHR relates to risks of all-cause death, stroke recurrence, and poor functional outcome at 3 months, 6 months, and 1 year of follow-up.

## 2 Materials and methods

### 2.1 Study approach and patient recruitment

Patients with AIS hospitalized at the Affiliated Brain Hospital of Nanjing Medical University within 1 week of symptom onset were enrolled in this ongoing, prospective, single-center, observational study. From January 2023 to December 2024, 901 consecutive patients were screened. The Ethics Review Committee approved the study (Ethics approval No. 2023-KY107-01).

**Figure 1:**
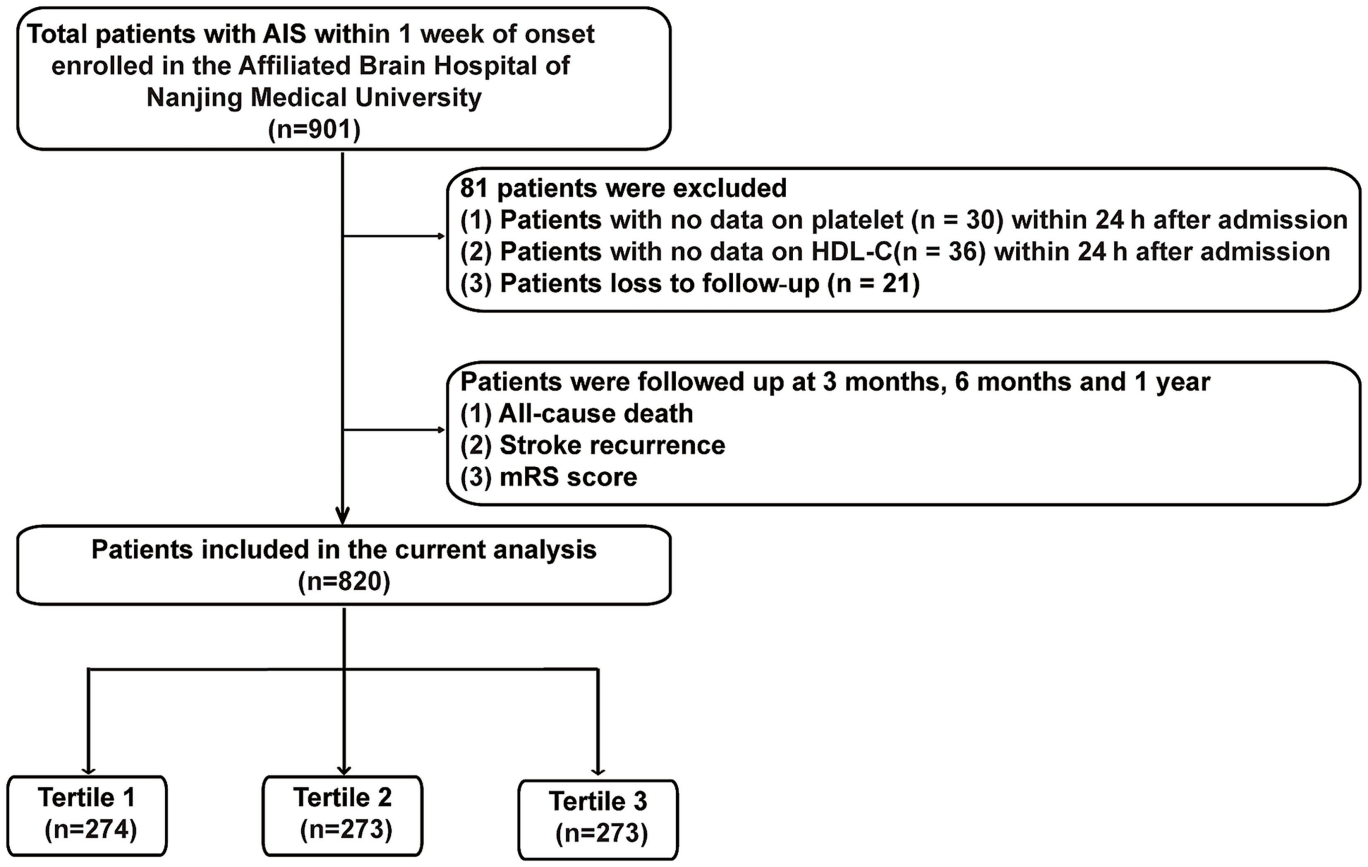
Flowchart of patient selection.

Figure 1 illustrates the study procedure. Exclusion criteria applied to the analysis were: (1) No PLT data at admission (*n* = 30); (2) No HDL-C data at admission (*n* = 36); (3) Loss to follow-up (*n* = 21). Finally, 820 patients were included.

### 2.2 Data collection and calculation

All patients were diagnosed by professional neurologists on the basis of neurological and radiological findings or carotid ultrasound. Examination and baseline data were documented by professional neurologists. Through direct patient interviews or medical record review, professional neurologists collected data: prestroke mRS score, NIHSS score at admission, and the stroke etiology as categorized by TOAST criteria (23). Medication use at admission was recorded and categorized into the following five groups: antiplatelet agents, anticoagulant agents, antihypertensive agents, hypoglycemic agents, and lipid-lowering agents.

### 2.3 Clinical laboratory examination

Fasting venous blood samples were extracted and collected in EDTA-K anticoagulant and non-anticoagulant vacuum tubes within 24 h after admission. Lipid profiles and platelet count (PC) were measured using the BECKMAN AU5820 automated biochemical analyzer and the Mindray BC-6800 automated hematology analyzer in the Clinical Laboratory. All laboratory measurements were conducted by technicians blinded to patients’ clinical information.

### 2.4 PHR

The PHR was calculated as the ratio of the PC (10^9^ cells/L) to HDL-C (mmol/L) (24). Because the normal range of PHR has not been well defined, we categorized patients into three groups on the tertiles of PHR: tertile 1, *<* 168.298, *n* = 274; tertile 2, 168.298–240, *n* = 273; and tertile 3, > 240, *n* = 273, with tertile 1 designated as the reference group for comparative analyses against tertile 2 and tertile 3. In addition to analyzing PHR as a continuous variable, this stratified approach allows for a granular evaluation of the associations of PHR with other variables or clinical outcomes.

### 2.5 Follow-up and assessment of clinical outcomes

Post-stroke clinical outcomes were assessed at 3-month, 6-month, and 1-year intervals. In the present study, clinical outcomes included all-cause death (defined as death from any cause), stroke recurrence (defined as a new ischemic or hemorrhagic stroke after symptom onset), and poor functional outcome. The primary definition of poor functional outcome was mRS 3–6 (moderate-to-severe disability or death). As a secondary analysis, we also examined mRS 2–6 (any disability or death) to assess the robustness of our findings; these results are presented in Supplementary Tables and Supplementary Figures. Trained researchers collected clinical outcomes via telephone or direct interviews with patients, relatives, or caregivers. Patients unreachable for 15 consecutive workdays were deemed lost to follow-up.

### 2.6 Assessments of covariates

Baseline demographic and clinical characteristics were meticulously collected. (1) Demographic variables included sex, age, educational level (primary or below, secondary, and tertiary or above), and current smoking and drinking status. (2) Body measurements included BMI, and blood pressure. (3) Clinical characteristics and in-hospital medications included medical history (hypertension, diabetes, dyslipidemia, coronary heart disease, atrial fibrillation, stroke, and cerebral hemorrhage), medications used during hospitalization (five categories), and other covariates including prestroke mRS score, time to admission, NIHSS score at admission, and TOAST classification. (4) Laboratory test data included PLT, total cholesterol (TC), triglycerides (TG), HDL-C, and low-density lipoprotein cholesterol (LDL-C).

### 2.7 Statistical analysis

Enrolled patients were classified into three groups according to PHR tertiles. In the final cohort of 820 patients, all baseline variables were completely collected. We used the Kolmogorov–Smirnov method to evaluate the distribution of baseline data. Non-normally distributed continuous data were expressed as medians with interquartile range (IQR). Group differences were tested using Kruskal-Wallis for continuous variables and chi-square or Fisher’s exact for categorical variables.

Kaplan–Meier survival curves and the log-rank test were used to evaluate the cumulative risks of all-cause death and stroke recurrence stratified by PHR tertiles. To examine the relationships between the PHR and the risks of all-cause death and stroke recurrence risk, we established both univariate and multivariate Cox regression models, and hazard ratios (HRs) with 95% confidence intervals (CIs) were reported. To examine the relationship between the PHR and the risk of poor functional outcome, we established both univariate and multivariate logistic regression models, and odds ratios (ORs) with 95% CI were reported. PHR was incorporated into the models as both a continuous variable and categorical variable. No violations of the proportional hazards assumption were detected using scaled Schoenfeld residuals. Variables with *p* < 0.100 in univariable analysis were selected for multivariable adjustment (Table 1). Multicollinearity was assessed using variance inflation factors (VIFs), and all variables with VIF < 3 were retained in the final multivariable models (Supplementary Table S1). We fitted an unadjusted model and three adjusted models (Models 1–3). Model 1 was adjusted for age and sex. Model 2 was adjusted for age, sex, educational level, BMI, hypertension, dyslipidemia, atrial fibrillation, current smoking status, time to admission, TOAST classification, anticoagulant agents, antihypertensive agents, and hypoglycemic agents. Model 3 was adjusted for the variables in Model 2 plus TG and LDL-C. To provide a more intuitive demonstration of the relationships between PHR and clinical outcomes, we performed RCS analysis and subgroup analysis after adjustment for the variables in Model 3. Model calibration was assessed using the Hosmer-Lemeshow test and calibration curves, while clinical net benefit was evaluated through decision curve analysis (DCA). In addition, to assess the incremental predictive efficacy of PHR when added to the basic model for clinical outcomes, we conducted C-statistic, IDI, and NRI analyses. Using IBM SPSS Statistics version 27.0.0 (IBM Corporation) and R version 4.5.1 for all analyses, statistical significance was defined as a two-tailed *p*-value< 0.05.

**Table 1:** Baseline characteristics according to tertiles of PHR.

Characteristics	Total	PHR			*p* value
		Tertile 1 < 168.298	Tertile 2 168.298–240	Tertile 3 > 240	
No. of the patients	820	274	273	273	
Age, median (IQR), years	68 (60–75)	70 (65–77)	69 (61–75)	65 (56–73)	< 0.001
Female, *n* (%)	284 (34.6)	107 (39.1)	101 (37.0)	76 (27.8)	0.014
Educational level, *n* (%)					0.054
Primary	274 (33.4)	108 (39.4)	79 (28.9)	87 (31.9)	
Secondary	444 (54.1)	130 (47.4)	157 (57.5)	157 (57.5)	
Third	102 (12.4)	36 (13.1)	37 (13.6)	29 (10.6)	
BMI, median (IQR), kg/m^2^	24.221 (22.309–26.629)	23.987 (21.718–25.952)	24.221 (22.354–26.573)	24.802 (22.773–27.006)	0.003
Medical history, *n* (%)					
Hypertension	608 (74.1)	185 (67.5)	204 (74.7)	219 (80.2)	0.003
Diabetes	296 (36.1)	89 (32.5)	103 (37.7)	104 (38.1)	0.310
Dyslipidemia	93 (11.3)	26 (9.5)	42 (15.4)	25 (9.2)	0.036
Coronary heart disease	72 (8.8)	25 (9.1)	24 (8.8)	23 (8.4)	0.959
Atrial fibrillation	48 (5.9)	27 (9.9)	8 (2.9)	13 (4.8)	0.002
Stroke	243 (29.6)	81 (29.6)	77 (28.2)	85 (31.1)	0.755
Cerebral hemorrhage	42 (5.1)	13 (4.7)	15 (5.5)	14 (5.1)	0.924
Current smoking and alcohol drinking status, n (%)					
Current smoking status	186 (22.7)	48 (17.5)	58 (21.2)	80 (29.3)	0.003
Current alcohol drinking status	137 (16.7)	49 (17.9)	46 (16.8)	42 (15.4)	0.734
Admission stroke data					
Time to admission, median (IQR), day	2 (0–4)	2 (0–4)	2 (0–4)	2 (1–4)	0.089
Time from admission to blood collection, median (IQR), hour	15 (12–19)	15 (11–19)	15 (13–19)	15 (13–19)	0.198
NIHSS at admission, median (IQR)	3 (1–6)	3 (1–6)	3 (1–5)	3 (2–6)	0.210
Prestroke mRS score 2–5, *n* (%)	59 (7.2)	15 (5.5)	21 (7.7)	23 (8.4)	0.381
Stroke etiology, *n* (%)					0.062
Large-artery atherosclerosis	387 (47.2)	119 (43.4)	128 (46.9)	140 (51.3)	
Cardioembolism	37 (4.5)	22 (8.0)	7 (2.6)	8 (2.9)	
Small-vessel occlusion	224 (27.3)	76 (27.7)	74 (27.1)	74 (27.1)	
Other determined etiology	6 (0.7)	1 (0.4)	3 (1.1)	2 (0.7)	
Undetermined etiology	166 (20.2)	56 (20.4)	61 (22.3)	49 (17.9)	
Blood pressure, median (IQR), mmHg					
Systolic pressure	140 (130–157)	140 (130–158)	140 (130–156)	140 (130–154)	0.445
Diastolic pressure	80.5 (78.5–90)	80 (77–90)	82 (80–90)	82 (80–91)	0.195
Treatment in hospital, *n* (%)					
Antiplatelet agents	747 (91.1)	244 (89.1)	255 (93.4)	248 (90.8)	0.199
Anticoagulant agents	121 (14.8)	59 (21.5)	24 (8.8)	38 (13.9)	< 0.001
Antihypertensive agents	518 (63.2)	159 (58.0)	173 (63.4)	186 (68.1)	0.050
Any hypoglycemic agents	262 (32.0)	74 (27.0)	93 (34.1)	95 (34.8)	0.097
Any lipid-lowering agents	729 (88.9)	242 (88.3)	244 (89.4)	243 (89.0)	0.923
rt-PA intravenous thrombolytic	39 (4.9)	18 (6.6)	13 (4.8)	8 (2.9)	0.136
Mechanical thrombectomy	2 (0.2)	1 (0.4)	0 (0.0)	1 (0.4)	0.999
Laboratory data, median (IQR)					
PLT, 10^9^ cells/L	198 (163–240)	152 (129–177)	202 (182–226)	251 (213–289)	< 0.001
TG, mmol/L	1.39 (1.05–1.955)	1.160 (0.920–1.650)	1.390 (1.070–1.870)	1.600 (1.200–2.390)	< 0.001
TC, mmol/L	4.245 (3.46–4.945)	4.245 (3.490–4.850)	4.310 (3.490–5.020)	4.070 (3.380–4.930)	0.318
LDL-C, mmol/L	2.29 (1.73–2.825)	2.190 (1.670–2.630)	2.400 (1.800–2.920)	2.290 (1.730–2.870)	0.009
HDL-C, mmol/L	0.97 (0.83–1.14)	1.150 (1.000–1.370)	0.990 (0.900–1.110)	0.820 (0.720–0.930)	< 0.001
PHR	202.155 (153.120–262.365)	136.197 (114.474–153.153)	202.198 (184.762–217.143)	286.275 (262.376–339.535)	-

Patients were divided into three groups according to the tertiles of PHR: tertile 1, < 168.298; tertile 2, 168.298–240; and tertile 3, > 240. PHR, platelet/high-density lipoprotein cholesterol ratio; HDL-C, high-density lipoprotein cholesterol; PLT, platelet; BMI, body mass index; IQR, interquartile range; mRS, modified Rankin Scale; NIHSS, the National Institutes of Health Stroke Scale; rt-PA, recombinant tissue plasminogen activator; LDL-C, low-density lipoprotein cholesterol; TC, total cholesterol; TG, triglyceride.

## 3 Results

### 3.1 Baseline characteristics

Among the 820 patients with AIS, the median age was 68 years (IQR, 60–75), and 34.6% were women. The median PHR in the study population was 202.155 (IQR, 153.120–262.365). Based on the tertiles of the PHR distribution, the study population was categorized into three groups. The median PHR levels in the three groups were 136.197 (IQR, 114.474–153.153), 202.198 (IQR, 184.762–217.143), and 286.275 (IQR, 262.376–339.535), respectively.

Baseline characteristics according to PHR tertiles are presented in Table 1. Patients in tertile 3 were more likely to be younger (*p <* 0.001), male (*p =* 0.014), have a higher BMI (*p =* 0.003) and be current smokers (*p =* 0.003). In addition, the prevalence of hypertension (*p =* 0.003) was higher and that of atrial fibrillation (*p =* 0.002) was lower in tertile 3 compared to tertile 1. Patients in tertile 3 were more likely to receive antihypertensive agents (*p =* 0.050) and less likely to receive anticoagulant agents (*p <* 0.001). Compared with tertile 1, the tertile 3 showed significantly higher levels of TG (*p <* 0.001) and LDL-C (*p* = 0.009). The remaining baseline variables did not differ significantly between tertiles 1 and 3.

### 3.2 Cumulative risks of all-cause death and stroke recurrence: analysis using Kaplan–Meier survival curves

**Figure 2:**
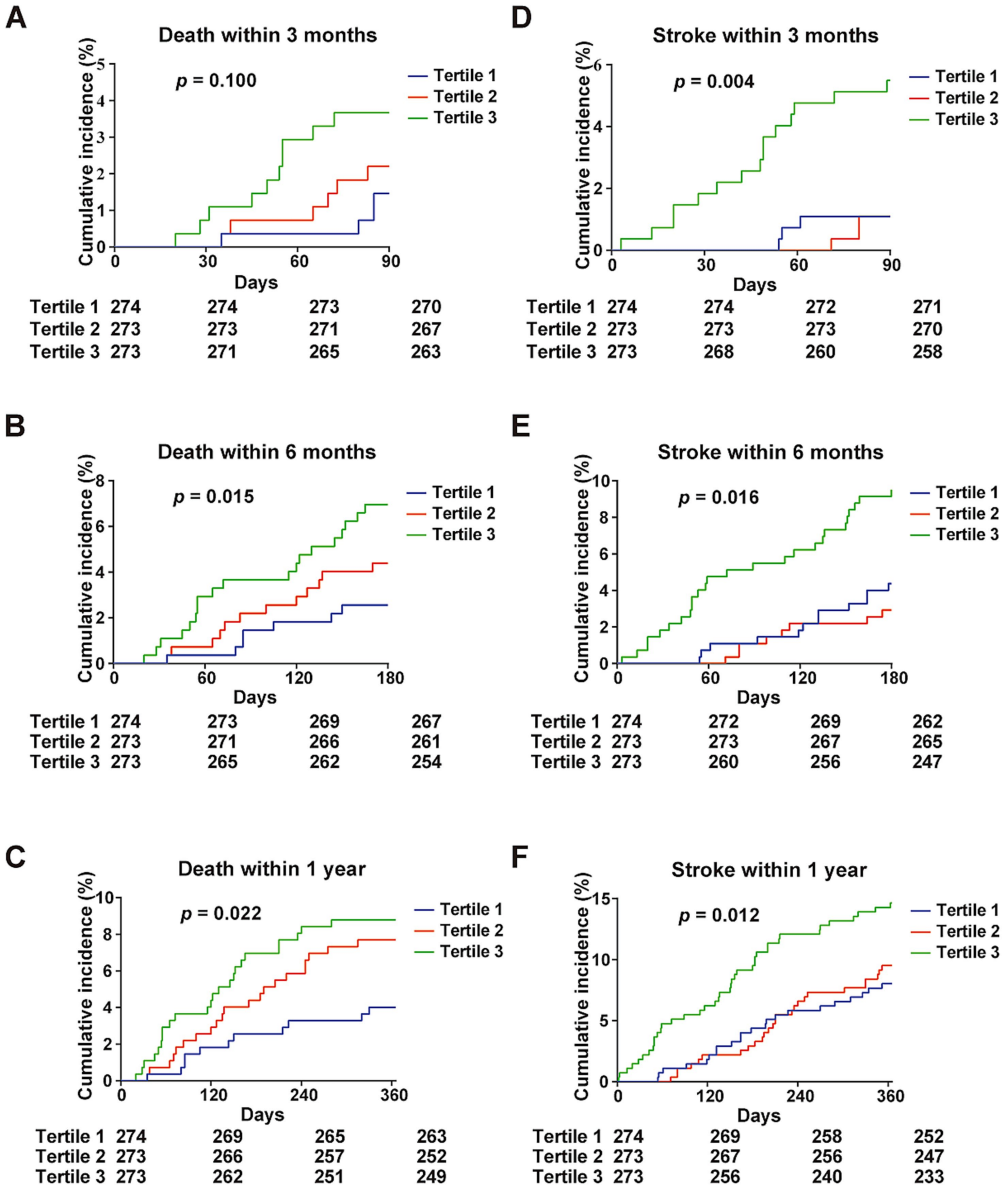
Kaplan–Meier survival curves. Cumulative risks of all-cause death at 3 months **(A)**, 6 months **(B)**, 1 year **(C)**, cumulative risks rates of stroke recurrence at 3 months **(D)**, 6 months **(E)**, 1 year **(F)**.

Among the 820 patients, 2.4% (*n* = 20) died at the 3 months follow-up, increasing to 4.6% (*n* = 38) at 6 months and 6.8% (*n* = 56) at 1 year (Table 2). Stroke recurrence occurred in 2.6% (*n* = 21) of patients by 3 months, increasing to 5.6% (*n* = 46) at 6 months and 10.7% (*n* = 88) at 1 year (Table 2). Figures 2A–C show that the cumulative risk of all-cause death increased in tertile 3; this trend was significant at 6 months (log-rank *p* = 0.015) and 1 year (log-rank *p* = 0.022), but not at 3 months (log-rank *p* = 0.100). Similarly, Figures 2D–F show that the cumulative risk of stroke recurrence also increased in tertile 3 and was significantly higher at 3 months (log-rank *p* = 0.004), 6 months (log-rank *p* = 0.016), and 1 year (log-rank *p* = 0.012).

**Table 2:** Associations of PHR with all-cause death, stroke recurrence, and poor functional outcome.

Outcome		PHR	Event, *n* (%)	Unadjusted HR/OR (95% CI)	*p* value	Model 1	*p* value	Model 2	*p* value	Model 3	*p* value
At 3 months											
	All-cause death										
		Tertile 1	4 (1.5)	1.00 (ref)	-	1.00 (ref)	-	1.00 (ref)	-	1.00 (ref)	-
		Tertile 2	6 (2.2)	1.514 (0.427–5.365)	0.521	1.746 (0.490–6.217)	0.39	2.146 (0.540–8.527)	0.278	2.120 (0.526–8.538)	0.291
		Tertile 3	10 (3.7)	2.557 (0.802–8.154)	0.112	3.272 (1.010–10.595)	0.048	4.903 (1.373–17.512)	0.014	5.008 (1.408–17.805)	0.013
		Per 1 SD increase		1.005 (1.002–1.008)	0.002	1.006 (1.003–1.009)	< 0.001	1.009 (1.004–1.013)	< 0.001	1.009 (1.004–1.013)	< 0.001
	Stroke										
		Tertile 1	3 (1.1)	1.00 (ref)	-	1.00 (ref)	-	1.00 (ref)	-	1.00 (ref)	-
		Tertile 2	3 (1.1)	1.001 (0.202–4.959)	0.999	0.995 (0.200–4.941)	0.995	1.217 (0.241–6.161)	0.812	1.197 (0.235–6.090)	0.829
		Tertile 3	15 (5.5)	5.161 (1.494–17.828)	0.009	4.977 (1.409–17.577)	0.013	6.992 (1.817–26.901)	0.005	6.789 (1.726–26.697)	0.006
		Per 1 SD increase		1.005 (1.001–1.008)	0.005	1.004 (1.001–1.008)	0.013	1.005 (1.001–1.009)	0.011	1.005 (1.001–1.009)	0.02
	mRS score 3–6										
		Tertile 1	48 (17.5)	1.00 (ref)	-	1.00 (ref)	-	1.00 (ref)	-	1.00 (ref)	-
		Tertile 2	53 (19.4)	1.134 (0.736–1.748)	0.568	1.190 (0.769–1.842)	0.434	1.334 (0.841–2.115)	0.221	1.310 (0.824–2.083)	0.253
		Tertile 3	72 (26.4)	1.687 (1.117–2.546)	0.013	1.860 (1.215–2.847)	0.004	1.912 (1.220–2.998)	0.005	1.672 (1.052–2.655)	0.03
		Per 1 SD increase		1.004 (1.002–1.006)	< 0.001	1.005 (1.003–1.007)	< 0.001	1.005 (1.002–1.007)	< 0.001	1.004 (1.002–1.006)	< 0.001
At 6 months											
	All-cause death										
		Tertile 1	7 (2.6)	1.00 (ref)	-	1.00 (ref)	-	1.00 (ref)	-	1.00 (ref)	-
		Tertile 2	12 (4.4)	1.737 (0.684–4.412)	0.246	1.902 (0.746–4.845)	0.178	2.254 (0.822–6.184)	0.114	2.333 (0.845–6.441)	0.102
		Tertile 3	19 (7.0)	2.793 (1.174–6.643)	0.02	3.350 (1.389–8.079)	0.007	4.196 (1.627–10.823)	0.003	4.285 (1.641–11.186)	0.003
		Per 1 SD increase		1.005 (1.003–1.007)	< 0.001	1.006 (1.004–1.008)	< 0.001	1.007 (1.004–1.010)	< 0.001	1.007 (1.004–1.010)	< 0.001
	Stroke										
		Tertile 1	12 (4.4)	1.00 (ref)	-	1.00 (ref)	-	1.00 (ref)	-	1.00 (ref)	-
		Tertile 2	8 (2.9)	0.667 (0.272–1.631)	0.374	0.670 (0.273–1.642)	0.381	0.715 (0.287–1.786)	0.473	0.707 (0.283–1.767)	0.458
		Tertile 3	26 (9.5)	2.263 (1.142–4.485)	0.019	2.238 (1.109–4.515)	0.024	2.363 (1.129–4.945)	0.022	2.063 (0.965–4.406)	0.062
		Per 1 SD increase		1.004 (1.001–1.006)	0.003	1.004 (1.001–1.006)	0.005	1.004 (1.001–1.006)	0.008	1.003 (1.000–1.006)	0.028
	mRS score 3–6										
		Tertile 1	40 (14.6)	1.00 (ref)	-	1.00 (ref)	-	1.00 (ref)	-	1.00 (ref)	-
		Tertile 2	50 (18.3)	1.312 (0.833–2.066)	0.242	1.393 (0.880–2.205)	0.157	1.534 (0.947–2.486)	0.082	1.510 (0.929–2.454)	0.097
		Tertile 3	69 (25.3)	1.979 (1.284–3.049)	0.002	2.242 (1.433–3.506)	< 0.001	2.279 (1.424–3.645)	< 0.001	1.978 (1.221–3.205)	0.006
		Per 1 SD increase		1.005 (1.003–1.007)	< 0.001	1.005 (1.003–1.007)	< 0.001	1.005 (1.003–1.007)	< 0.001	1.005 (1.003–1.007)	< 0.001
At 1 year											
	All-cause death										
		Tertile 1	11 (4.0)	1.00 (ref)	-	1.00 (ref)	-	1.00 (ref)	-	1.00 (ref)	-
		Tertile 2	21 (7.7)	1.949 (0.940–4.042)	0.073	2.073 (0.997–4.312)	0.051	2.352 (1.089–5.080)	0.029	2.451 (1.128–5.326)	0.024
		Tertile 3	24 (8.8)	2.264 (1.109–4.622)	0.025	2.537 (1.226–5.251)	0.012	2.869 (1.336–6.160)	0.007	2.787 (1.278–6.078)	0.01
		Per 1 SD increase		1.005 (1.003–1.007)	< 0.001	1.005 (1.003–1.007)	< 0.001	1.006 (1.003–1.008)	< 0.001	1.005 (1.003–1.008)	< 0.001
	Stroke										
		Tertile 1	22 (8.0)	1.00 (ref)	-	1.00 (ref)	-	1.00 (ref)	-	1.00 (ref)	-
		Tertile 2	26 (9.5)	1.185 (0.672–2.091)	0.558	1.194 (0.676–2.111)	0.541	1.259 (0.704–2.252)	0.437	1.256 (0.701–2.250)	0.443
		Tertile 3	40 (14.7)	1.926 (1.145–3.240)	0.014	1.938 (1.138–3.300)	0.015	1.926 (1.113–3.333)	0.019	1.682 (0.958–2.954)	0.070
		Per 1 SD increase		1.003 (1.001–1.005)	0.002	1.003 (1.001–1.005)	0.003	1.003 (1.001–1.005)	0.005	1.002 (1.000–1.005)	0.026
	mRS score 3–6										
		Tertile 1	37 (13.5)	1.00 (ref)	-	1.00 (ref)	-	1.00 (ref)	-	1.00 (ref)	-
		Tertile 2	43 (15.8)	1.198 (0.744–1.927)	0.457	1.263 (0.782–2.040)	0.339	1.360 (0.823–2.245)	0.23	1.338 (0.809–2.214)	0.257
		Tertile 3	66 (24.2)	2.042 (1.311–3.183)	0.002	2.294 (1.451–3.626)	< 0.001	2.369 (1.466–3.827)	< 0.001	2.140 (1.309–3.498)	0.002
		Per 1 SD increase		1.005 (1.003–1.007)	< 0.001	1.006 (1.004–1.008)	< 0.001	1.006 (1.004–1.008)	< 0.001	1.005 (1.003–1.008)	< 0.001

HRs with 95% CIs were used for all-cause death and stroke recurrence; ORs with 95% CIs were used for mRS scores 3–6. Model 1: adjusted for age and sex. Model 2: adjusted for age, sex, educational level, BMI, hypertension, dyslipidemia, atrial fibrillation, current smoking, time to admission, stroke etiology, anticoagulant agents, antihypertensive agents, and hypoglycemic agents. Model 3: adjusted for variables in model 2, plus TG and LDL-C. PHR, platelet/high-density lipoprotein cholesterol ratio; HR, hazard ratio; OR, odds ratio; mRS, modified Rankin Scale; SD, standard deviation.

### 3.3 Cox regression models for assessing the relationships between PHR and all-cause death and stroke recurrence

To explore the independent relationships between PHR and the risks of all-cause death and stroke recurrence, we constructed Cox regression models (Table 2). When PHR was analyzed as a continuous variable, each 1-unit increase was significantly associated with a higher risk of all-cause death (crude HR = 1.005; 95% CI 1.002–1.008 at 3 months; crude HR = 1.005; 95% CI 1.003–1.007 at 6 months; crude HR = 1.005; 95% CI 1.003–1.007 at 1 year) and stroke recurrence (crude HR = 1.005; 95% CI 1.001–1.008 at 3 months; crude HR = 1.004; 95% CI 1.001–1.006 at 6 months; crude HR = 1.003; 95% CI 1.001–1.005 at 1 year) in the unadjusted model (all *p* < 0.05). These associations persisted after further adjustment for potential confounders in Models 1–3 (all *p* < 0.05). PHR tertiles also showed a progressively increasing trend in the risks of all-cause death and stroke recurrence across the unadjusted and adjusted models. In the unadjusted model, tertile 3 was positively associated with all-cause death (crude HR = 2.557; 95% CI 0.802–8.154; *p* = 0.112 at 3 months; crude HR = 2.793; 95% CI 1.174–6.643; *p* = 0.020 at 6 months; crude HR = 2.264; 95% CI 1.109–4.622; *p* = 0.025 at 1 year) and stroke recurrence (crude HR = 5.161; 95% CI 1.494–17.828; *p* = 0.009 at 3 months; crude HR = 2.263; 95% CI 1.142–4.485; *p* = 0.019 at 6 months; crude HR = 1.926; 95% CI 1.145–3.240; *p* = 0.014 at 1 year) compared with tertile 1. These positive associations were further confirmed in Models 1 and 2 (all *p* < 0.05). After full adjustment in Model 3, the positive association between PHR and the risk of all-cause death remained significant (adjusted HR = 5.008; 95% CI 1.408–17.805; *p* = 0.013 at 3 months; adjusted HR = 4.285; 95% CI 1.641–11.186; *p* = 0.003 at 6 months; adjusted HR = 2.787; 95% CI 1.278–6.078; *p* = 0.010 at 1 year). For stroke recurrence, the association remained numerically positive in Model 3 (adjusted HR = 6.789; 95% CI 1.726–26.697; *p* = 0.006 at 3 months; adjusted HR = 2.063; 95% CI 0.965–4.406; *p* = 0.062 at 6 months; adjusted HR = 1.682; 95% CI 0.958–2.954; *p* = 0.070 at 1 year).

### 3.4 Logistic regression models for assessing the relationship between PHR and poor functional outcome

Among the 820 patients, the rate of poor functional outcome (mRS 3–6) was 21.1% (*n* = 173) at 3 months, 19.4% (*n* = 159) at 6 months, and 17.8% (*n* = 146) at 1 year (Table 2). To explore the independent relationship between PHR and the risk of mRS 3–6, we constructed logistic regression models (Table 2). When PHR was analyzed as a continuous variable, each 1-unit increase was significantly associated with a higher risk of mRS 3–6 (crude OR = 1.004; 95% CI 1.002–1.006 at 3 months; crude OR = 1.005; 95% CI 1.003–1.007 at 6 months; crude OR = 1.005; 95% CI 1.003–1.007 at 1 year) in the unadjusted model (all *p* < 0.05). This association persisted after further adjustment for potential confounders in Models 1–3 (all *p* < 0.05). PHR tertiles demonstrated a significantly increasing trend in the risk of poor functional outcome (mRS 3–6) across all unadjusted and adjusted models (Table 2). In the unadjusted model, tertile 3 was positively associated with mRS 3–6 (crude OR = 1.687; 95% CI 1.117–2.546; *p* = 0.013 at 3 months; crude OR = 1.979; 95% CI 1.284–3.049; *p* = 0.002 at 6 months; crude OR = 2.042; 95% CI 1.311–3.183; *p* = 0.002 at 1 year) compared with tertile 1. These positive associations were further confirmed in Models 1 and 2 (all *p* < 0.05). After full adjustment in Model 3, the positive association between PHR and the risk of mRS 3–6 remained significant (adjusted OR = 1.672; 95% CI 1.052–2.655; *p* = 0.030 at 3 months; adjusted OR = 1.978; 95% CI 1.221–3.205; *p* = 0.006 at 6 months; adjusted OR = 2.140; 95% CI 1.309–3.498; *p* = 0.002 at 1 year). Similar findings for mRS 2–6 are shown in Supplementary Table S2 (all *p* < 0.05).

### 3.5 Analysis of relationships among PHR and clinical outcomes risk using RCS models and subgroup analysis

**Figure 3:**
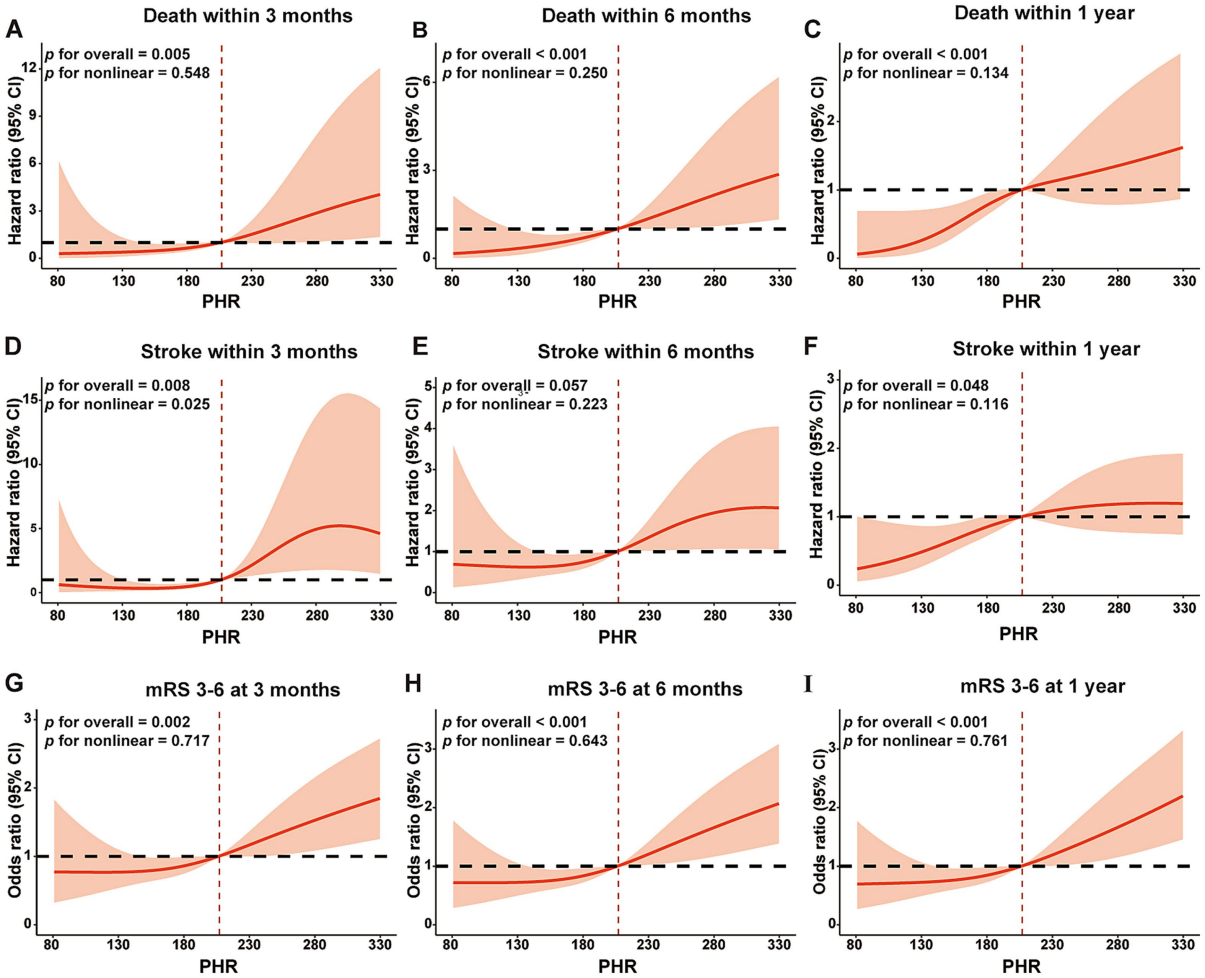
RCS models analyzing the relationship between PHR and clinical outcomes. All-cause death at 3 months **(A)**, 6 months **(B)**, and 1 year **(C)**; stroke recurrence at 3 months **(D)**, 6 months **(E)**, and 1 year **(F)**; poor functional outcome (mRS 3-6) at 3 months **(G)**, 6 months **(H)**, and 1 year **(I)**. Adjusted for age, sex, educational level, BMI, hypertension, dyslipidemia, atrial fibrillation, current smoking, time to admission, TOAST classification, anticoagulant agents, antihypertensive agents, hypoglycemic agents, TG, and LDL-C.

In the RCS analysis, we further explored potential nonlinear relationships between PHR and clinical outcomes in patients with AIS. Figures 3A–C presented adjusted RCS models showing a positive linear relationship between PHR and the risk of all-cause death at 3 months, 6 months and 1 year (all *p* for nonlinear > 0.05). Figures 3G–I and Supplementary Figures S1A–C showed similar findings for the risks of mRS 3–6 and mRS 2–6 at 3 months, 6 months, and 1 year (all *p* for nonlinear > 0.05). Figures 3D–F show that, in the overall AIS population, PHR was positively and linearly correlated with the risk of stroke recurrence at 6 months and 1 year (all *p* for nonlinear > 0.05), but not at 3 months (*p* for nonlinear = 0.025). Based on the RCS-derived cutoff value of 206.14 (PHR corresponding to HR/OR = 1), PHR levels above this threshold were associated with HRs/ORs > 1 across all time points and clinical outcomes, suggesting that PHR > 206.14 may serve as a reference threshold for increased risk of adverse outcomes in AIS patients (Figure 3; Supplementary Figure S1). To further explore the associations between PHR and clinical outcome risk, we performed subgroup analyses stratified by age, sex, BMI, NIHSS score at admission, and current smoking status (Supplementary Figures S2–S5). Supplementary Figure S2 shows the relationship between PHR and all-cause death in each subgroup at 3 months, 6 months, and 1 year. Except for age (interaction *p* < 0.05), the interactions between PHR and the other stratified variables were not statistically significant (interaction *p* > 0.05). In addition, subgroup analysis of PHR and stroke recurrence showed no significant interactions with subgroup variables (*p* for interaction > 0.05; Supplementary Figure S3). Supplementary Figure S4 shows the relationship between PHR and mRS 3–6 in each subgroup at 3 months, 6 months, and 1 year. Except for BMI (interaction *p* < 0.05), the interactions between PHR and the other stratified variables were not statistically significant (interaction *p* > 0.05). Furthermore, subgroup analysis of PHR and mRS 2–6 at 3 months, 6 months, and 1 year (Supplementary Figure S5) showed no significant interaction between subgroup variables except BMI at 1 year (*p* for interaction < 0.05).

### 3.6 Model performance and incremental predictive values of PHR in patients with AIS

We first evaluated the basic model (adjusted for age, sex, educational level, BMI, hypertension, dyslipidemia, atrial fibrillation, current smoking status, time to admission, TOAST classification, anticoagulant agents, antihypertensive agents, hypoglycemic agents, TG, and LDL-C) calibration using the Hosmer-Lemeshow goodness-of-fit test. For all outcomes across each time point, the resulting *p*-values exceeded 0.05, demonstrating no substantial lack of fit and confirming satisfactory model calibration (Supplementary Table S4).

We further examined whether adding PHR to the basic model could improve the predictive power for all-cause death, stroke recurrence, and poor functional outcome (mRS 3–6) in patients with AIS during follow-up periods of 3 months, 6 months, and 1 year. The basic model incorporating PHR also showed good calibration (*p* > 0.05; Supplementary Table S4). As summarized in Table 3, adding PHR to the basic model significantly improved the C-statistic, IDI, and NRI for all-cause death and poor functional outcome (mRS 3–6) at 6 months, and 1 year (all *p* < 0.05). For all-cause death, PHR did not improve the C-statistic, continuous NRI, or IDI at 3 months (all *p* > 0.05). For mRS 3–6, PHR improved the continuous NRI (*p <* 0.05) and IDI (*p <* 0.05) at 3 months, but not the C-statistic (*p* = 0.055). Similar results were found for mRS 2–6 in Supplementary Table S2. For stroke recurrence, adding PHR significantly increased only the C-statistic at 1 year (*p* > 0.05 at 3 and 6 months). DCA showed that both the basic model and the full model (Basic model + PHR) provided positive net benefit across clinically relevant risk thresholds, indicating their potential clinical applicability. The DCA results were consistent with the findings from C-statistic, NRI, and IDI, further supporting the incremental value of adding PHR where statistical improvements were observed (Supplementary Figure S6).

**Table 3:** Performance of models with PHR to predict all-cause death, stroke recurrence and poor functional outcome.

Model	C-statistic		IDI		Continuous NRI	
	Estimate (95% CI)	*p* value	Estimate (95% CI)	*p* value	Estimate (95% CI)	*p* value
At 3 months						
Death						
Basic model	0.846 (0.770–0.923)	Reference	Reference	-	Reference	-
Basic model + PHR	0.874 (0.803–0.944)	0.252	0.043 (−0.012–0.098)	0.126	0.122 (−0.070–0.314)	0.214
Stroke recurrence						
Basic model	0.795 (0.694–0.896)	Reference	Reference	-	Reference	-
Basic model + PHR	0.813 (0.722–0.904)	0.243	0.003 (−0.004–0.010)	0.370	0.050 (−0.042–0.142)	0.285
mRS score 3–6						
Basic model	0.682 (0.638–0.726)	Reference	Reference	-	Reference	-
Basic model + PHR	0.704 (0.662–0.746)	0.055	0.018 (0.005–0.030)	0.006	0.297 (0.131–0.463)	< 0.001
At 6 months						
Death						
Basic model	0.738 (0.655–0.820)	Reference	Reference	-	Reference	-
Basic model + PHR	0.802 (0.736–0.868)	0.008	0.053 (0.009–0.096)	0.017	0.181 (0.015–0.348)	0.033
Stroke recurrence						
Basic model	0.669 (0.590–0.749)	Reference	Reference		Reference	
Basic model + PHR	0.698 (0.627–0.768)	0.079	0.000 (−0.001–0.002)	0.518	−0.013 (−0.089–0.063)	0.738
mRS score 3–6						
Basic model	0.675 (0.629–0.720)	Reference	Reference		Reference	
Basic model + PHR	0.709 (0.666–0.752)	0.011	0.025 (0.009–0.040)	0.002	0.379 (0.208–0.550)	< 0.001
At 1 year						
Death						
Basic model	0.696 (0.621–0.771)	Reference	Reference		Reference	
Basic model + PHR	0.745 (0.676–0.813)	0.043	0.089 (0.038–0.140)	0.001	0.300 (0.136–0.464)	< 0.001
Stroke recurrence						
Basic model	0.611 (0.542–0.679)	Reference	Reference		Reference	
Basic model + PHR	0.640 (0.577–0.702)	0.028	0.001 (−0.003–0.005)	0.542	0.018 (−0.015–0.050)	0.291
mRS score 3–6						
Basic model	0.651 (0.602–0.700)	Reference	Reference		Reference	
Basic model + PHR	0.696 (0.650–0.742)	0.011	0.033 (0.015–0.050)	< 0.001	0.396 (0.219–0.573)	< 0.001

Basic model included adjusted for age, sex, educational level, BMI, hypertension, dyslipidemia, atrial fibrillation, current smoking, time to admission, stroke etiology, anticoagulant agents, antihypertensive agents, hypoglycemic agents, TG, and LDL-C. PHR, platelet/high-density lipoprotein cholesterol ratio; mRS, modified Rankin Scale; CI, confidence interval; C-statistic, concordance statistic; IDI, integrated discrimination improvement; NRI, net reclassification index.

## 4 Discussion

Based on this cohort study, we demonstrated a significant positive association between PHR and the risk of all-cause death, stroke recurrence, and poor functional outcome in patients with AIS at 3 months, 6 months and 1 year of follow-up. After adjustment for the potential confounders, a higher PHR level remained associated with a higher risk of adverse clinical outcomes. RCS analysis demonstrated a positive dose–response relationship between PHR and clinical outcomes. Subgroup analysis showed that the association between PHR and all-cause death varied by age and that the association between PHR and poor functional outcome (mRS 3–6) varied by BMI. The risk of all-cause death was more pronounced in patients aged ≥ 60 years (*p* for interaction < 0.05) and the risk of mRS 3–6 was significantly stronger in patients with BMI ≥  24 kg/m^2^ (*p* for interaction < 0.05) at 3 months, 6 months and 1 year, indicating that older age and higher body weight may enhance the adverse impact of PHR on all-cause death and mRS 3–6. C-statistic, continuous NRI, and IDI analyses indicated that PHR may be a valuable predictor of short- and long-term prognosis in patients with AIS. These findings support the potential clinical application of PHR in outcome assessment and may help identify high-risk populations and guide more effective risk management strategies.

AIS represents a complex clinical entity that arises when blood flow to a cerebral territory is disrupted (1, 25). Regardless of the underlying mechanism, AIS often results in significant neurological deficits and death (26, 27). Therefore, with continuing advances in AIS treatment, it is critically important to identify indicators with predictive value (1).

PHR, as an emerging biomarker, has been proposed for assessing hypercoagulability and metabolic disorder status (28). Previous research has suggested a potential link between PHR and an increased prevalence of cognitive decline in older adults, metabolic syndrome (MetS), depression, and CAD in type 2 diabetes mellitus (T2DM) (20, 21, 29, 30). Moreover, PHR has been suggested by previous research to be a novel prognostic marker for patients with CVDs. A population-based investigation demonstrated that in middle-aged and elderly people, more severe coronary artery lesions are associated with higher PHR levels (17). Results from the NHANES suggested that the CVD mortality among stroke survivors increased in a positive linear manner as PHR levels rose (31). Adverse long-term clinical outcomes in CAD patients, whether or not they had T2DM, were shown to be related to elevated PHR according to a real-world observational cohort analysis (32). According to a study that utilized the CHARLS data, individuals with higher PHR levels had a significantly increased risk of stroke, and this same metric may be useful for the early detection of high-risk subgroups. However, evidence regarding the relationship between PHR and the short- and long-term prognosis of AIS was limited. Our study confirmed the associations between PHR and all-cause death, stroke recurrence, and poor functional outcome in patients with AIS during short- and long-term follow-up. In addition, we further demonstrated the predictive value of PHR for clinical outcomes after AIS using C-statistics, IDI, and NRI.

Platelet aggregation, aberrant platelet regulation, and atherosclerotic pathology may collectively contribute to the potential association observed between PHR and clinical outcomes. Elevated PHR reflects the coexistence of platelet aggregation and lipid metabolism disorders. Studies have shown that PLTs are an important component of thrombi in stroke (16). Cerebral reperfusion injury, arising from the integration of thrombotic and inflammatory processes in stroke, often manifests as platelet activation and aggregation plus platelet-immune cell crosstalk, all of which contribute to the deterioration of microvascular function (33, 34). Circulating immune cells have been shown to induce immunothrombosis and actively participate in thrombus formation by promoting platelet recruitment and thrombin activation (35, 36). During ischemic stroke, cerebral ischemia–reperfusion induces cyclophilin D (CypD)-mediated necrosis of platelets, and the subsequent crosstalk between these necrotic platelets and neutrophils contributes to the exacerbation of brain injury (37). Research using a rat model of ischemic stroke has demonstrated that cerebral dopamine neurotrophic factor (CDNF) significantly suppressed platelet activation and aggregation, curtailed the production of lipid mediators, limited infarct volume, and lessened neurological deficits (33, 38). C-C motif chemokine ligand 21 (CCL21) enhances platelet activation and atherothrombosis by binding to platelet C-C motif chemokine receptor 7 (CCR7), thereby activating downstream inhibitory G protein (Gi) and G13 signaling pathways (39). Given the links between metabolic disease and atherogenic dyslipidemia, lower HDL-C levels are considered a risk factor for ischemic stroke (40). HDL-C exerts its main atheroprotective actions by promoting reverse cholesterol transport and inhibiting inflammatory responses, therefore, reduced HDL-C levels impair reverse cholesterol transport and aggravate atherosclerosis (41, 42). An analysis that pooled data from six large prospective cohort studies indicated that HDL-C levels below 50 mg/dL may be associated with an elevated risk for both ischemic and hemorrhagic stroke (43). A study of 429,759 UK Biobank participants showed that both extremely low and extremely high HDL-C levels increased the risk of death from stroke through different mechanisms (44). In addition, thromboinflammation and reduced HDL-C may increase the instability of atherosclerotic plaques (35, 45). Hence, focusing on PHR can provide valuable clinical insights for preventing and delaying the occurrence and progression of AIS.

In the subgroup analysis, PHR showed significant interactions with all-cause death in patients aged ≥  60 years and with mRS 3–6 in patients with BMI ≥   24 kg/m^2^. In particular, age-related endothelial cell inflammation can provoke vascular dysfunction and contribute to the pathogenesis of cerebrovascular disease, including ischemic stroke (46). In aged mice, ischemic brain microvascular occlusion by neutrophils was increased after experimental stroke, together with elevated oxidative stress, enhanced phagocytosis, and heightened procoagulant features, resulting in poorer reperfusion and prognosis compared with young mice (47). Physical activity provides a protective effect against obesity, T2DM, and other cardiometabolic diseases such as stroke (48, 49). One experimental study demonstrated that pre-stroke pharmacological targeting of obesity through glucagon-like peptide 1 receptor (GLP-1R)/neuropeptide Y receptor Y2 (NPY2R) activation in T2DM enhanced stroke recovery (49). Neutrophil α9 expression was more significantly increased in obese mice after stroke and was accompanied by larger brain infarcts, increased post-reperfusion thromboinflammation, poorer cerebral blood flow, and worse long-term functional outcomes (50). Hyperglycemia primes platelet hyperactivity and procoagulant platelet formation, thereby exacerbating ischemic stroke outcome (51). Another study suggested that dietary intervention to achieve weight loss in obese mice can enhance post-stroke functional recovery, an effect associated with the normalization of glucose metabolism and the reduction of stroke-induced inflammation (52). These changes may promote hypercoagulability and lipid metabolism disorders, thereby increasing PHR levels. Future studies should further investigate the age-specific and sex-specific physiological mechanisms underlying the impact of PHR on all-cause death and mRS 3–6.

This study has several notable strengths. First, we acknowledge that similar lipid-related ratios have been studied in stroke populations (53, 54). However, to our knowledge, it is the first prospective cohort study specifically focused on patients with AIS to explore the relationship between PHR and the prognostic values of all-cause death, stroke recurrence, and poor functional outcome with long-term follow-up (3 months, 6 months, and 1 year). Second, the robustness of our findings is reinforced by three factors: the study’s relatively long follow-up duration, its comprehensive approach to measuring potential covariates, and its reliable procedures for outcome assessment. Third, PHR is easy to obtain in clinical practice, which increases its practical utility. Finally, we performed subgroup analyses across different population characteristics, which provides valuable insights for clinical practice.

However, several limitations should also be recognized. First, the sample was derived from a single-center cohort with a relatively homogeneous Chinese population. Differences in racial composition, living environment, diet, and other variables may hinder the extrapolation of the present findings to other settings, as potential racial, ethnic, and socioeconomic differences were not accounted for. External validation in larger, multi-center, and multi-ethnic cohorts is therefore needed. Second, residual or unmeasured confounding may have been introduced. Therefore, future research should incorporate multicenter and cross-cultural studies to validate the broader applicability of our findings. Third, the sample size was relatively small, and the incidence of some clinical outcomes was relatively low, which may limit statistical power and make it difficult to clarify the association between PHR and individual components of the primary endpoint. Fourth, because PHR was assessed exclusively at baseline and not reassessed during subsequent follow-up, the potential effect of its temporal variation on clinical outcomes cannot be ascertained. This may have underestimated risk and failed to reflect the impact of dynamic metabolic state changes on outcomes. Future studies should investigate how longitudinal changes in PHR influence clinical outcomes. Fifth, the diagnosis of stroke recurrence was primarily based on whether patients had a history of readmission to our hospital or other hospitals after discharge, as confirmed by medical diagnosis records obtained through telephone follow-up inquiries. We acknowledge that this approach may introduce potential bias or underdiagnosis, as patients who experienced a recurrent stroke but did not seek medical attention or were admitted to hospitals outside our follow-up network may have been missed. For mRS assessment, trained researchers conducted structured interviews with patients, relatives, or caregivers via telephone. When responses were unclear or uncertain, we further performed video calls to verify the patients’ functional status, minimizing potential misclassification. Nonetheless, some degree of information bias is inherent to telephone-based follow-up, which remains a common constraint in large epidemiological studies (55). Finally, given the observational design of this investigation, drawing causal conclusions about the relationship between PHR and clinical endpoints is not possible. Further mechanistic research and clinical trials are required to solidify the evidence.

## 5 Conclusion

Our research results indicate that PHR is not only cost-effective but also potentially clinically valuable for assessing the all-cause death, stroke recurrence and poor functional outcome among patients with AIS at 3 months, 6 months and 1 year follow-up. This study supports the potential of PHR as a predictive biomarker for risk stratification of all-cause death, stroke recurrence and poor functional outcome in patients with AIS.

## Data Availability

The raw data supporting the conclusions of this article will be made available by the authors, without undue reservation.
