# Supplementary figures and images for: Platelet to high-density lipoprotein cholesterol ratio predicts clinical outcomes after acute ischemic stroke: a prospective cohort study
Source: Front Neurol. 2026 Jun 30;17:1851022. doi: 10.3389/fneur.2026.1851022 (PMC13364632; doi:10.3389/fneur.2026.1851022)

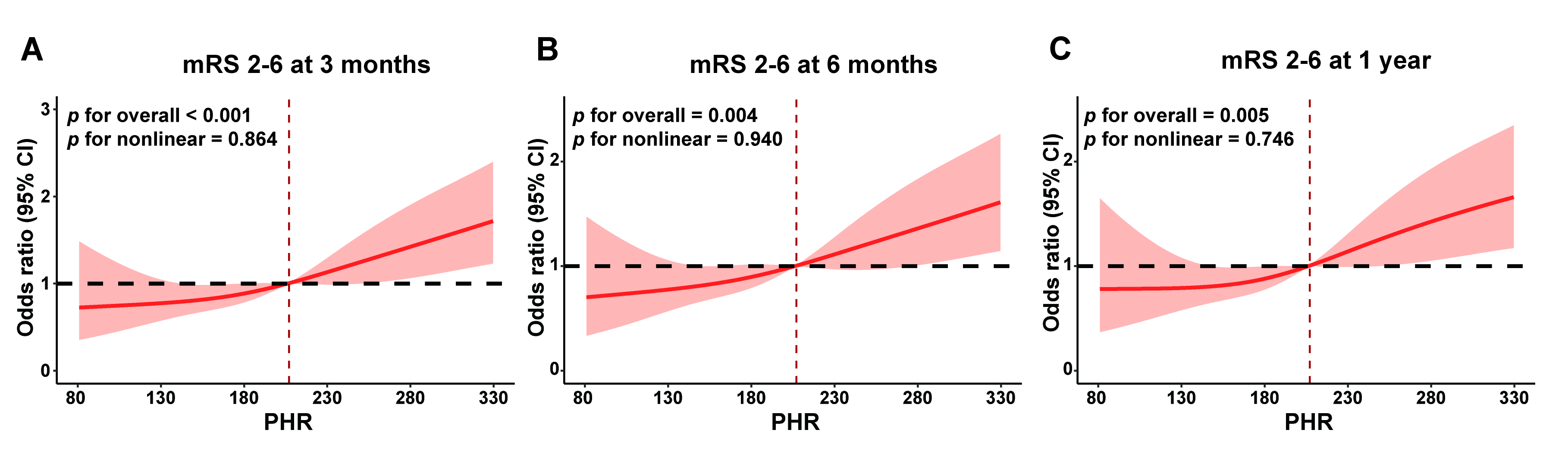

Supplement: Supplementary file 1 [file Image_1.TIF]

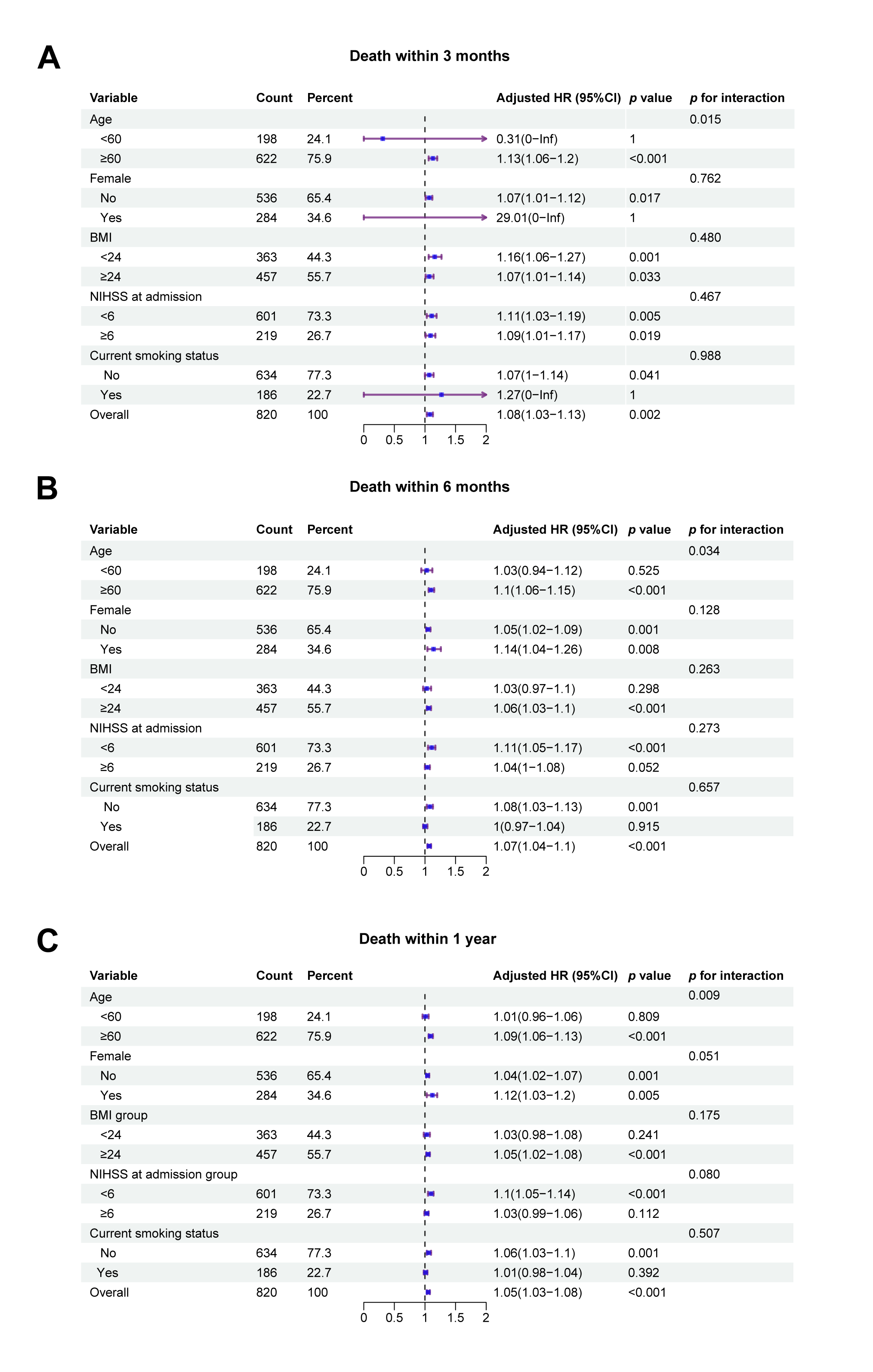

Supplement: Supplementary file 2 [file Image_2.TIF]

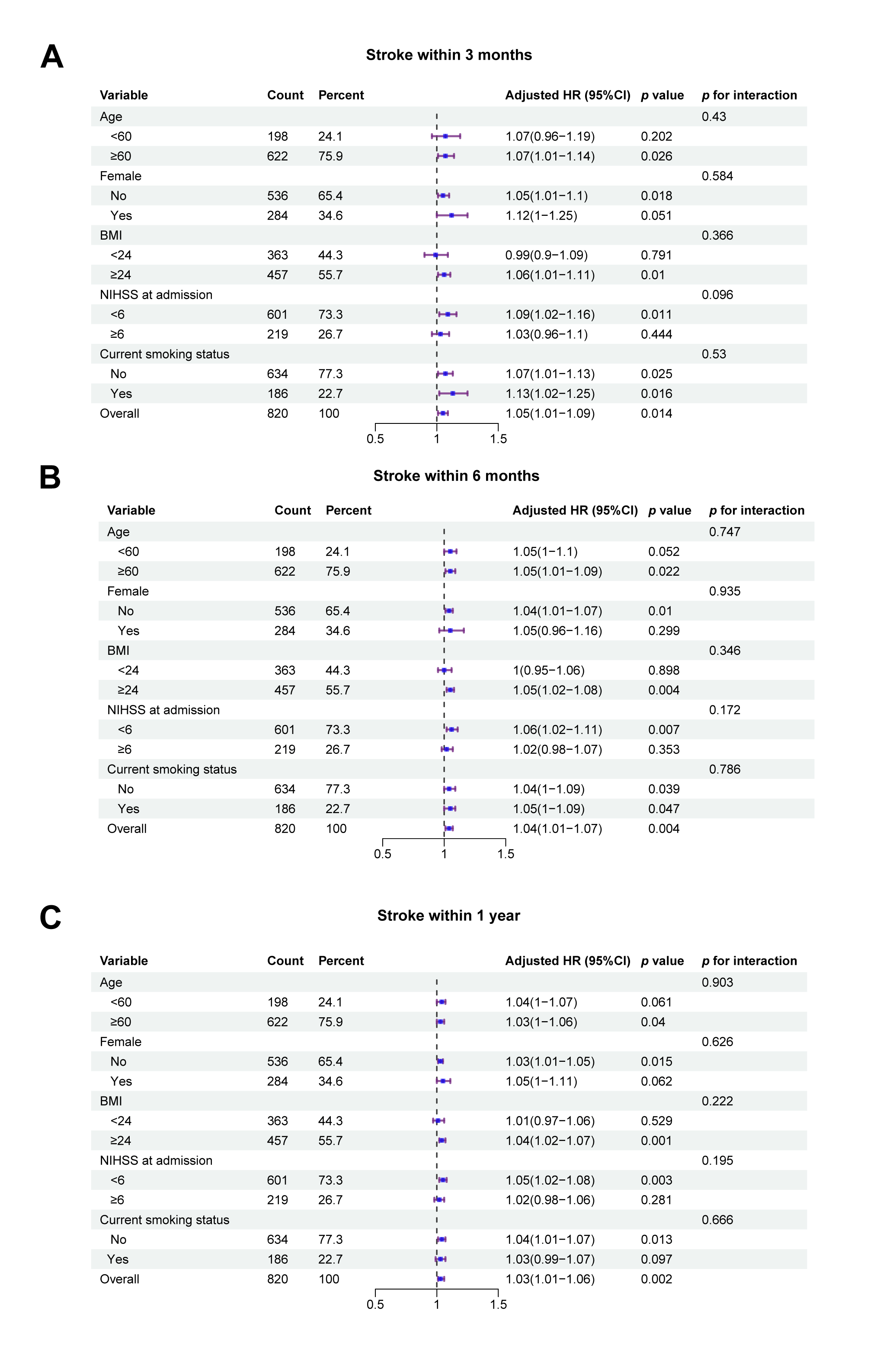

Supplement: Supplementary file 3 [file Image_3.TIF]

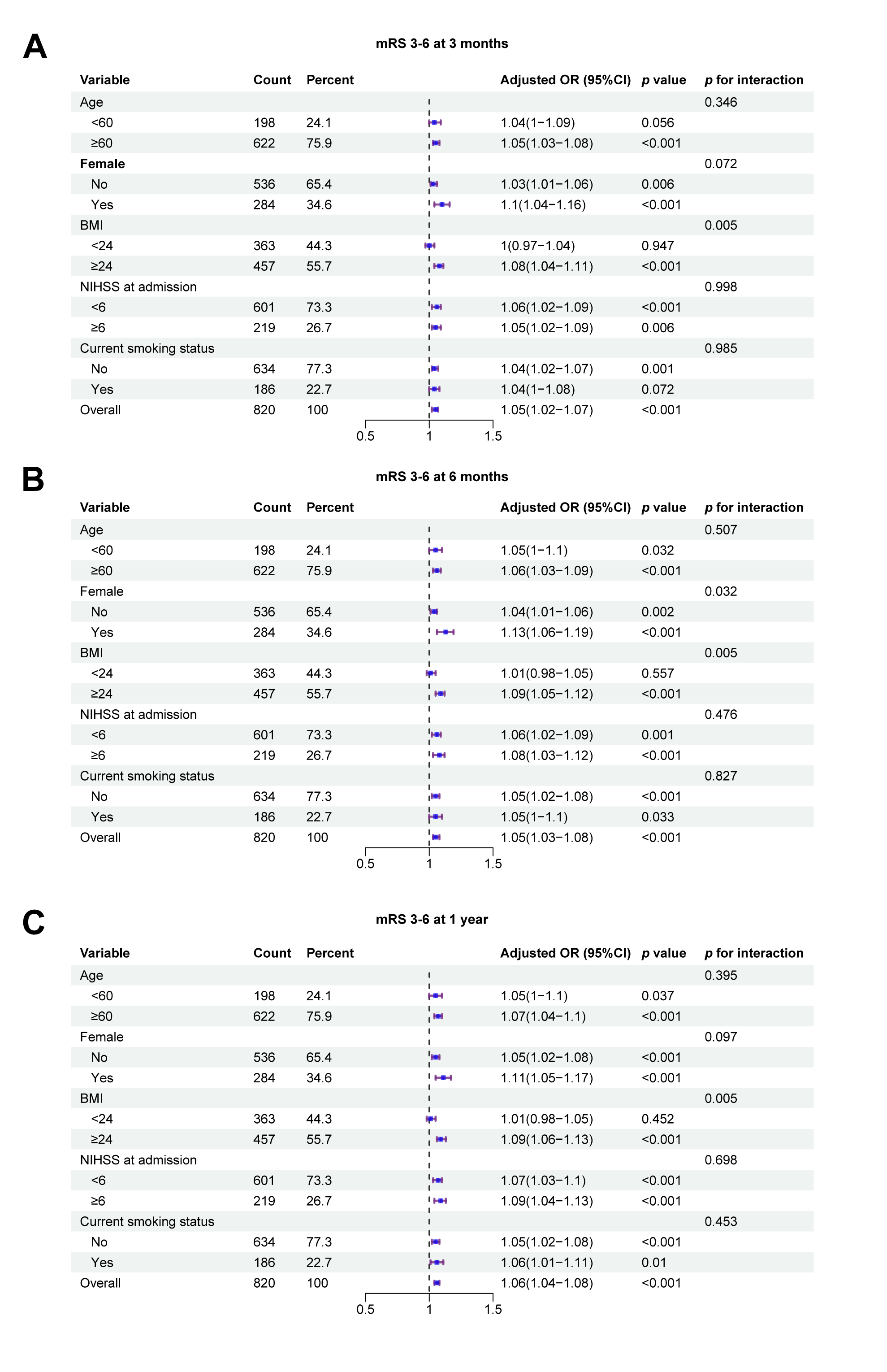

Supplement: Supplementary file 4 [file Image_4.TIF]

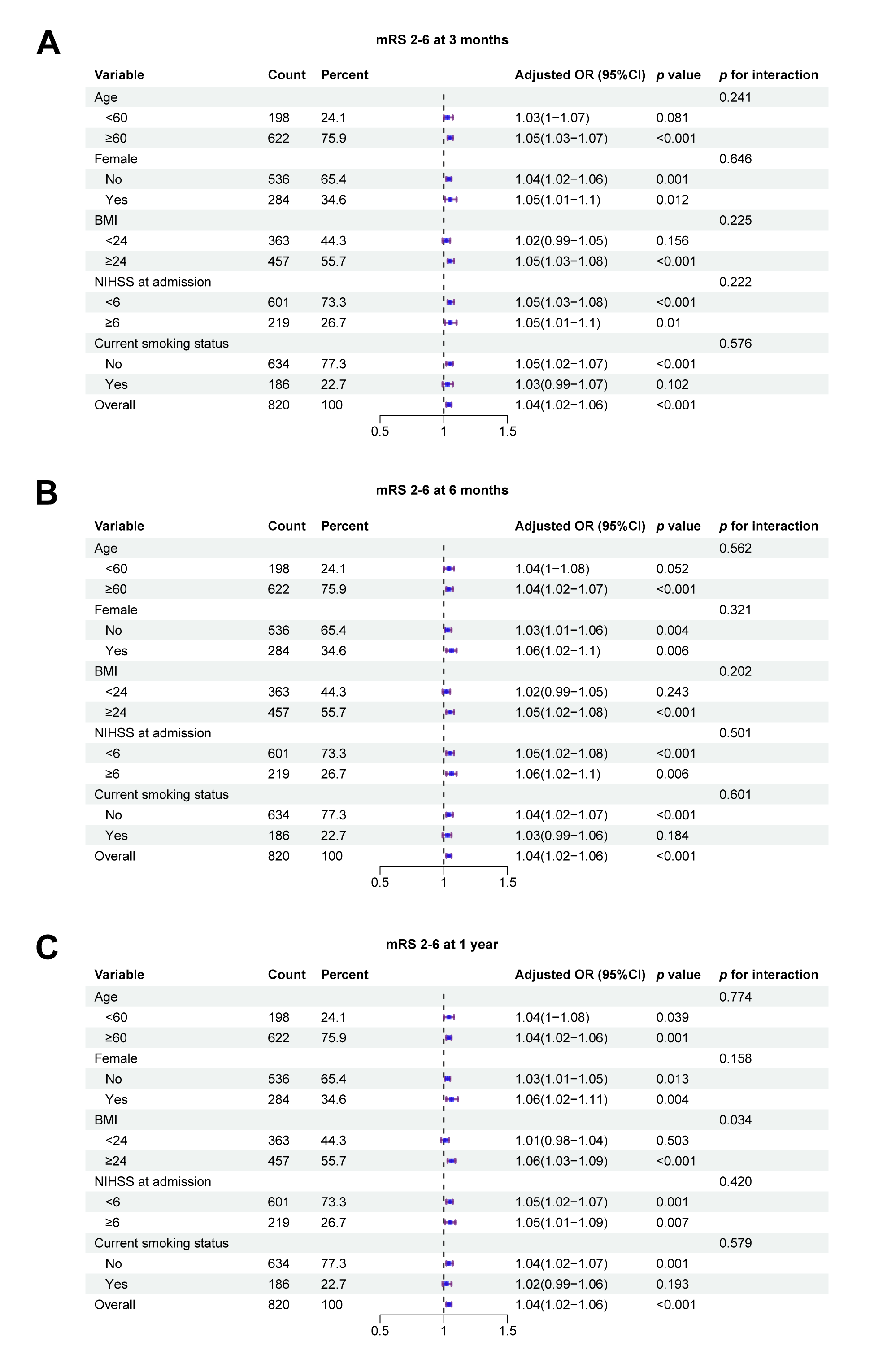

Supplement: Supplementary file 5 [file Image_5.TIF]

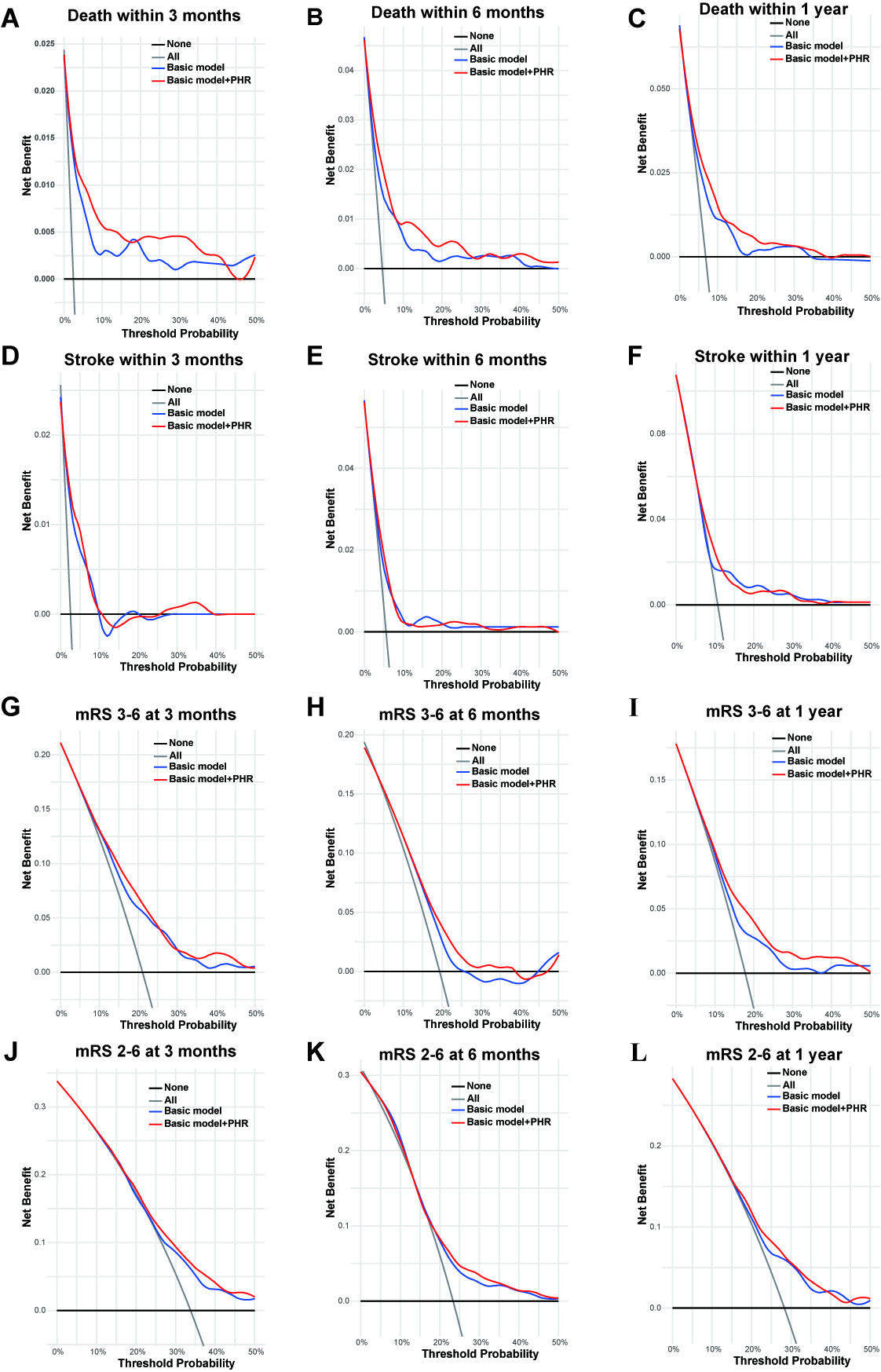

Supplement: Supplementary file 6 [file Image_6.TIF]
