# Supplementary material for: Platelet to high-density lipoprotein cholesterol ratio predicts clinical outcomes after acute ischemic stroke: a prospective cohort study
Source: Front Neurol. 2026 Jun 30;17:1851022. doi: 10.3389/fneur.2026.1851022 (PMC13364632; doi:10.3389/fneur.2026.1851022)
Supplement: Supplementary file 7 [file Supplementary_file_1.docx]

**Supplementary material**

**Supplementary figure legends**

**Figure S1. RCS models analyzing the relationship between PHR and mRS 2-6.**

Adjusted for age, sex, educational level, BMI, hypertension, dyslipidemia, atrial fibrillation, current smoking, time to admission, TOAST classification, anticoagulant agents, antihypertensive agents, hypoglycemic agents, TG, and LDL-C. (A) 3 months. (B) 6 months. (C) 1 year.

**Figure S2. Forest plots of subgroup analyses for the association between PHR and all-cause death.**

Adjusted for age, sex, educational level, BMI, hypertension, dyslipidemia, atrial fibrillation, current smoking, time to admission, TOAST classification, anticoagulant agents, antihypertensive agents, hypoglycemic agents, TG, and LDL-C except for the covariate that was stratified. (A) 3 months. (B) 6 months. (C) 1 year.

**Figure S3. Forest plots of subgroup analyses for the association between PHR and stroke recurrence.**

Adjusted for age, sex, educational level, BMI, hypertension, dyslipidemia, atrial fibrillation, current smoking, time to admission, TOAST classification, anticoagulant agents, antihypertensive agents, hypoglycemic agents, TG, and LDL-C except for the covariate that was stratified. (A) 3 months. (B) 6 months. (C) 1 year.

**Figure S4. Forest plots of subgroup analyses for the association between PHR and mRS 3-6.**

Adjusted for age, sex, educational level, BMI, hypertension, dyslipidemia, atrial fibrillation, current smoking, time to admission, TOAST classification, anticoagulant agents, antihypertensive agents, hypoglycemic agents, TG, and LDL-C except for the covariate that was stratified. (A) 3 months. (B) 6 months. (C) 1 year.

**Figure S5. Forest plots of subgroup analyses for the association between PHR and mRS 2-6.**

Adjusted for age, sex, educational level, BMI, hypertension, dyslipidemia, atrial fibrillation, current smoking, time to admission, TOAST classification, anticoagulant agents, antihypertensive agents, hypoglycemic agents, TG, and LDL-C except for the covariate that was stratified. (A) 3 months. (B) 6 months. (C) 1 year.

**Figure S6. DCA curves for all clinical outcomes at 3 months, 6 months, and 1 year.**

DCA curves were generated to evaluate the clinical net benefit of the basic model and the basic model incorporating PHR. Basic model was adjusted for age, sex, educational level, BMI, hypertension, dyslipidemia, atrial fibrillation, current smoking, time to admission, stroke etiology, anticoagulant agents, antihypertensive agents, and hypoglycemic agents, TG, and LDL-C. (A) 3 months. (B) 6 months. (C) 1 year.

**Supplementary tables**

**Table S1 Variance inflation factor and tolerance**

| **Variables** | **Tolerance** | **VIF** |
| --- | --- | --- |
| Age | 0.821 | 1.219 |
| Female | 0.815 | 1.228 |
| Educational level | 0.924 | 1.083 |
| BMI | 0.912 | 1.096 |
| Hypertension | 0.453 | 2.207 |
| Dyslipidemia | 0.874 | 1.144 |
| Atrial fibrillation | 0.761 | 1.314 |
| Current smoking status | 0.826 | 1.211 |
| Time to admission | 0.944 | 1.059 |
| Stroke etiology | 0.967 | 1.034 |
| Anticoagulant agents | 0.78 | 1.282 |
| Antihypertensive agents | 0.436 | 2.293 |
| Any hypoglycemic agents | 0.928 | 1.077 |
| TG | 0.808 | 1.237 |
| LDL-C | 0.901 | 1.11 |
| PHR | 0.856 | 1.168 |

Abbreviations: PHR, platelet/high-density lipoprotein cholesterol ratio; VIF: variance inflation factor; BMI, body mass index; LDL-C, low-density lipoprotein cholesterol; TG, triglyceride.

**Table S2. Associations of PHR with poor functional outcome defined as mRS 2-6.**

| **Outcome** | **PHR** | **Event, n (%)** | **Unadjusted OR (95% CI)** | ***p* value** | **Model 1** | ***p* value** | **Model 2** | ***p* value** | **Model 3** | ***p* value** |
| --- | --- | --- | --- | --- | --- | --- | --- | --- | --- | --- |
| mRS score 2-6 at 3 months | | | | | | | | | | |
|  | Tertile 1 | 79 (28.8) | 1.00 (ref) | - | 1.00 (ref) | - | 1.00 (ref) | - | 1.00 (ref) | - |
|  | Tertile 2 | 86 (31.5) | 1.135 (0.788-1.636) | 0.496 | 1.181 (0.817-1.707) | 0.376 | 1.235 (0.841-1.814) | 0.281 | 1.199 (0.814-1.765) | 0.358 |
|  | Tertile 3 | 112 (41.0) | 1.717 (1.203-2.451) | 0.003 | 1.877 (1.300-2.710) | < 0.001 | 1.891 (1.288-2.777) | 0.001 | 1.685 (1.134-2.504) | 0.01 |
|  | Per 1 SD increase | | 1.004 (1.002-1.006) | < 0.001 | 1.004 (1.003-1.006) | < 0.001 | 1.004 (1.002-1.006) | < 0.001 | 1.004 (1.002-1.006) | < 0.001 |
| mRS score 2-6 at 6 months | | | | | | | | | | |
|  | Tertile 1 | 72 (26.3) | 1.00 (ref) | - | 1.00 (ref) | - | 1.00 (ref) | - | 1.00 (ref) | - |
|  | Tertile 2 | 79 (28.9) | 1.142 (0.785-1.663) | 0.487 | 1.207 (0.826-1.763) | 0.332 | 1.207 (0.826-1.763) | 0.332 | 1.212 (0.815-1.802) | 0.343 |
|  | Tertile 3 | 99 (36.3) | 1.596 (1.108-2.300) | 0.012 | 1.806 (1.237-2.636) | 0.002 | 1.806 (1.237-2.636) | 0.002 | 1.555 (1.036-2.333) | 0.033 |
|  | Per 1 SD increase | | 1.003 (1.002-1.005) | < 0.001 | 1.004 (1.002-1.006) | < 0.001 | 1.004 (1.002-1.006) | < 0.001 | 1.004 (1.002-1.005) | < 0.001 |
| mRS score 2-6 at 1 year | | | | | | | | | | |
|  | Tertile 1 | 68 (24.8) | 1.00 (ref) | - | 1.00 (ref) | - | 1.00 (ref) | - | 1.00 (ref) | - |
|  | Tertile 2 | 70 (25.6) | 1.045 (0.710-1.537) | 0.825 | 1.105 (0.749-1.632) | 0.614 | 1.166 (0.779-1.746) | 0.456 | 1.140 (0.760-1.712) | 0.526 |
|  | Tertile 3 | 94 (34.4) | 1.591 (1.098-2.305) | 0.014 | 1.822 (1.241-2.675) | 0.002 | 1.817 (1.219-2.709) | 0.003 | 1.588 (1.052-2.397) | 0.028 |
|  | Per 1 SD increase | | 1.003 (1.002-1.005) | < 0.001 | 1.004 (1.002-1.006) | < 0.001 | 1.004 (1.002-1.006) | < 0.001 | 1.003 (1.001-1.005) | < 0.001 |

Note: ORs with 95% CIs were used for mRS scores 2-6.

Model 1: adjusted for age and sex.

Model 2: adjusted for age, sex, educational level, BMI, hypertension, dyslipidemia, atrial fibrillation, current smoking, time to admission, stroke etiology, anticoagulant agents, antihypertensive agents, and hypoglycemic agents.

Model 3: adjusted for variables in model 2, plus TG and LDL-C.

Abbreviations: PHR, platelet/high-density lipoprotein cholesterol ratio; ORs, odds ratio; mRS, modified Rankin Scale; SD, standard deviation.

**Table S3. Associations of PHR with all-cause death, stroke recurrence, and poor functional outcome.**

| **Outcome** | | **PHR** | **Adjusted HR/OR (95% CI) - Model 4** | ***p* value** |
| --- | --- | --- | --- | --- |
| At 3 months | | |  |  |
|  | All-cause death | |  |  |
|  |  | Tertile 1 | 1.00 (ref) | - |
|  |  | Tertile 2 | 2.298 (0.563-9.372) | 0.246 |
|  |  | Tertile 3 | 4.990 (1.380-18.049) | 0.014 |
|  |  | Per 1 SD increase | 1.008 (1.004-1.013) | < 0.001 |
|  | Stroke | |  |  |
|  |  | Tertile 1 | 1.00 (ref) | - |
|  |  | Tertile 2 | 1.297 (0.247-6.810) | 0.758 |
|  |  | Tertile 3 | 8.575 (1.992-36.909) | 0.004 |
|  |  | Per 1 SD increase | 1.005 (1.001-1.009) | 0.015 |
|  | mRS score 3-6 | |  |  |
|  |  | Tertile 1 | 1.00 (ref) | - |
|  |  | Tertile 2 | 1.597 (0.961-2.654) | 0.071 |
|  |  | Tertile 3 | 2.034 (1.210-3.418) | 0.007 |
|  |  | Per 1 SD increase | 1.005 (1.002-1.007) | < 0.001 |
|  | mRS score 2-6 | |  |  |
|  |  | Tertile 1 | 1.00 (ref) | - |
|  |  | Tertile 2 | 1.407 (0.917-2.161) | 0.118 |
|  |  | Tertile 3 | 2.091 (1.340-3.262) | 0.001 |
|  |  | Per 1 SD increase | 1.005 (1.003-1.007) | < 0.001 |
| At 6 months | | |  |  |
|  | All-cause death | |  |  |
|  |  | Tertile 1 | 1.00 (ref) | - |
|  |  | Tertile 2 | 2.653 (0.953-7.391) | 0.062 |
|  |  | Tertile 3 | 4.596 (1.721-12.277) | 0.002 |
|  |  | Per 1 SD increase | 1.007 (1.004-1.009) | < 0.001 |
|  | Stroke | |  |  |
|  |  | Tertile 1 | 1.00 (ref) | - |
|  |  | Tertile 2 | 0.714 (0.286-1.782) | 0.47 |
|  |  | Tertile 3 | 2.049 (0.957-4.386) | 0.065 |
|  |  | Per 1 SD increase | 1.003 (1.000-1.006) | 0.041 |
|  | mRS score 3-6 | |  |  |
|  |  | Tertile 1 | 1.00 (ref) | - |
|  |  | Tertile 2 | 1.810 (1.070-3.061) | 0.027 |
|  |  | Tertile 3 | 2.385 (1.400-4.065) | 0.001 |
|  |  | Per 1 SD increase | 1.005 (1.003-1.008) | < 0.001 |
|  | mRS score 2-6 | |  |  |
|  |  | Tertile 1 | 1.00 (ref) | - |
|  |  | Tertile 2 | 1.424 (0.921-2.202) | 0.112 |
|  |  | Tertile 3 | 1.839 (1.169-2.893) | 0.008 |
|  |  | Per 1 SD increase | 1.004 (1.002-1.006) | < 0.001 |
| At 1 year | | |  |  |
|  | All-cause death | |  |  |
|  |  | Tertile 1 | 1.00 (ref) | - |
|  |  | Tertile 2 | 2.592 (1.191-5.638) | 0.016 |
|  |  | Tertile 3 | 2.870 (1.304-6.316) | 0.009 |
|  |  | Per 1 SD increase | 1.005 (1.003-1.007) | < 0.001 |
|  | Stroke | |  |  |
|  |  | Tertile 1 | 1.00 (ref) | - |
|  |  | Tertile 2 | 1.283 (0.716-2.300) | 0.403 |
|  |  | Tertile 3 | 1.701 (0.967-2.994) | 0.065 |
|  |  | Per 1 SD increase | 1.003 (1.000-1.005) | 0.024 |
|  | mRS score 3-6 | |  |  |
|  |  | Tertile 1 | 1.00 (ref) | - |
|  |  | Tertile 2 | 1.565 (0.906-2.702) | 0.108 |
|  |  | Tertile 3 | 2.583 (1.492-4.472) | < 0.001 |
|  |  | Per 1 SD increase | 1.006 (1.004-1.009) | < 0.001 |
|  | mRS score 2-6 | |  |  |
|  |  | Tertile 1 | 1.00 (ref) | - |
|  |  | Tertile 2 | 1.311 (0.845-2.032) | 0.227 |
|  |  | Tertile 3 | 1.830 (1.164-2.876) | 0.009 |
|  |  | Per 1 SD increase | 1.004 (1.002-1.006) | < 0.001 |

Note: HRs with 95% CIs were used for all-cause death and stroke recurrence; ORs with 95% CIs were used for mRS scores 3-6.

Model 3: adjusted for age, sex, educational level, BMI, hypertension, dyslipidemia, atrial fibrillation, current smoking, time to admission, stroke etiology, anticoagulant agents, antihypertensive agents, hypoglycemic agents, TG, and LDL-C.

Model 4: adjusted for variables in model 3, plus key stroke-related predictors (*p* > 0.100), including NIHSS score at admission, rt-PA thrombolysis, and mechanical thrombectomy. In this extended model (Model 4), the direction and significance of the associations between PHR and clinical outcomes remained unchanged compared with Model 3.

Abbreviations: PHR, platelet/high-density lipoprotein cholesterol ratio; HR, hazard ratio; OR, odds ratio; mRS, modified Rankin Scale; SD, standard deviation.

**Table S4. Calibration performance of models for predicting clinical outcomes.**

| **Model** | **Hosmer-Lemeshow χ²** | **Hosmer-Lemeshow *p*** | **Calibration** |
| --- | --- | --- | --- |
| At 3 months | | | |
| Death | | | |
| Basic model | 6.98 | 0.5384 | Good |
| Basic model + PHR | 2.61 | 0.9562 | Good |
| Stroke recurrence | | | |
| Basic model | 6.81 | 0.557 | Good |
| Basic model + PHR | 7.43 | 0.4909 | Good |
| mRS score 3-6 | | | |
| Basic model | 8.02 | 0.4316 | Good |
| Basic model + PHR | 8.41 | 0.3949 | Good |
| mRS score 2-6 | | | |
| Basic model | 5.58 | 0.694 | Good |
| Basic model + PHR | 6.18 | 0.6271 | Good |
| At 6 months | | | |
| Death | | | |
| Basic model | 11.19 | 0.1912 | Good |
| Basic model + PHR | 6.36 | 0.6069 | Good |
| Stroke recurrence | | | |
| Basic model | 8.9 | 0.3511 | Good |
| Basic model + PHR | 7.34 | 0.4999 | Good |
| mRS score 3-6 | | | |
| Basic model | 8.31 | 0.4042 | Good |
| Basic model + PHR | 5.46 | 0.708 | Good |
| mRS score 2-6 | | | |
| Basic model | 5.06 | 0.7517 | Good |
| Basic model + PHR | 6.72 | 0.5668 | Good |
| At 1 year | | | |
| Death | | | |
| Basic model | 4.24 | 0.8349 | Good |
| Basic model + PHR | 2.50 | 0.9619 | Good |
| Stroke recurrence | | | |
| Basic model | 10.8 | 0.2131 | Good |
| Basic model + PHR | 6.37 | 0.6054 | Good |
| mRS score 3-6 | | | |
| Basic model | 12.38 | 0.1349 | Good |
| Basic model + PHR | 5.98 | 0.6496 | Good |
| mRS score 2-6 | | | |
| Basic model | 14.8 | 0.0632 | Good |
| Basic model + PHR | 9.03 | 0.34 | Good |

Basic model included adjusted for age, sex, educational level, BMI, hypertension, dyslipidemia, atrial fibrillation, current smoking, time to admission, stroke etiology, anticoagulant agents, antihypertensive agents, hypoglycemic agents, TG, and LDL-C.

Abbreviations: PHR, platelet/high-density lipoprotein cholesterol ratio; mRS, modified Rankin Scale.

**Table S5. Performance of models with PHR to predict poor functional outcome defined as mRS of 2-6.**

| **Model** | **C-statistic** | | **IDI** | | **Continuous NRI** | |
| --- | --- | --- | --- | --- | --- | --- |
|  | **Estimate (95% CI)** | ***p* value** | **Estimate (95% CI)** | ***p* value** | **Estimate (95% CI)** | ***p* value** |
| mRS score 2-6 at 3 months | | | | | | |
| Basic model | 0.637 (0.597-0.677) | Reference | Reference |  | Reference |  |
| Basic model+PHR | 0.663 (0.624-0.701) | 0.038 | 0.019 (0.008-0.029) | < 0.001 | 0.256 (0.113-0.399) | < 0.001 |
| mRS score 2-6 at 6 months | | | | | | |
| Basic model | 0.637 (0.596-0.679) | Reference | Reference |  | Reference |  |
| Basic model+PHR | 0.660 (0.620-0.700) | 0.046 | 0.015 (0.005-0.025) | 0.003 | 0.290 (0.143-0.437) | < 0.001 |
| mRS score 2-6 at 1 year | | | | | | |
| Basic model | 0.635 (0.592-0.677) | Reference | Reference |  | Reference |  |
| Basic model+PHR | 0.655 (0.614-0.697) | 0.071 | 0.014 (0.005-0.024) | 0.004 | 0.286 (0.136-0.436) | < 0.001 |

Basic model included adjusted for age, sex, educational level, BMI, hypertension, dyslipidemia, atrial fibrillation, current smoking, time to admission, stroke etiology, anticoagulant agents, antihypertensive agents, hypoglycemic agents, TG, and LDL-C.

Abbreviations: PHR, platelet/high-density lipoprotein cholesterol ratio; mRS, modified Rankin Scale; CI, confidence interval; C-statistic, concordance statistic; IDI, integrated discrimination improvement; NRI, net reclassification index.
